# Bacterial Community- and Hospital-Acquired Pneumonia in Patients with Critical COVID-19—A Prospective Monocentric Cohort Study

**DOI:** 10.3390/antibiotics13020192

**Published:** 2024-02-16

**Authors:** Lenka Doubravská, Miroslava Htoutou Sedláková, Kateřina Fišerová, Olga Klementová, Radovan Turek, Kateřina Langová, Milan Kolář

**Affiliations:** 1 Department of Anaesthesiology, Resuscitation and Intensive Care, University Hospital Olomouc, Zdravotniku 248/7, 779 00 Olomouc, Czech Republic; 2 Department of Anaesthesiology, Resuscitation and Intensive Care, Faculty of Medicine and Dentistry, Palacky University Olomouc, Hnevotinska 3, 779 00 Olomouc, Czech Republic; 3 Department of Microbiology, University Hospital Olomouc, Zdravotniku 248/7, 779 00 Olomouc, Czech Republicmilan.kolar@fnol.cz (M.K.); 4 Department of Microbiology, Faculty of Medicine and Dentistry, Palacky University Olomouc, Hnevotinska 3, 779 00 Olomouc, Czech Republic; 5 Department of Medical Biophysics, Faculty of Medicine and Dentistry, Palacky University Olomouc, Hnevotinska 3, 779 00 Olomouc, Czech Republic

**Keywords:** severe acute respiratory syndrome coronavirus-2 (SARS-CoV-2), critical coronavirus disease 19 (COVID-19), adult respiratory distress syndrome (ARDS), bacterial pneumonia, community-acquired pneumonia (CAP), hospital-acquired pneumonia (HAP), bacterial co- or superinfection, etiological agents, intensive care unit (ICU), mortality

## Abstract

The impact of bacterial pneumonia on patients with COVID-19 infection remains unclear. This prospective observational monocentric cohort study aims to determine the incidence of bacterial community- and hospital-acquired pneumonia (CAP and HAP) and its effect on mortality in critically ill COVID-19 patients admitted to the intensive care unit (ICU) at University Hospital Olomouc between 1 November 2020 and 31 December 2022. The secondary objectives of this study include identifying the bacterial etiology of CAP and HAP and exploring the capabilities of diagnostic tools, with a focus on inflammatory biomarkers. Data were collected from the electronic information hospital system, encompassing biomarkers, microbiological findings, and daily visit records, and subsequently evaluated by ICU physicians and clinical microbiologists. Out of 171 patients suffering from critical COVID-19, 46 (27%) had CAP, while 78 (46%) developed HAP. Critically ill COVID-19 patients who experienced bacterial CAP and HAP exhibited higher mortality compared to COVID-19 patients without any bacterial infection, with rates of 38% and 56% versus 11%, respectively. In CAP, the most frequent causative agents were chlamydophila and mycoplasma; Enterobacterales, which were multidrug-resistant in 71% of cases; Gram-negative non-fermenting rods; and *Staphylococcus aureus*. Notably, no strains of *Streptococcus pneumoniae* were detected, and only a single strain each of *Haemophilus influenzae* and *Moraxella catarrhalis* was isolated. The most frequent etiologic agents causing HAP were Enterobacterales and Gram-negative non-fermenting rods. Based on the presented results, commonly used biochemical markers demonstrated poor predictive and diagnostic accuracy. To confirm the diagnosis of bacterial CAP in our patient cohort, it was necessary to assess the initial values of inflammatory markers (particularly procalcitonin), consider clinical signs indicative of bacterial infection, and/or rely on positive microbiological findings. For HAP diagnostics, it was appropriate to conduct regular detailed clinical examinations (with a focus on evaluating respiratory functions) and closely monitor the dynamics of inflammatory markers (preferably Interleukin-6).

## 1. Introduction

Following the emergence of the COVID-19 pandemic caused by the SARS-CoV-2 virus, it became evident that a significant new pathogen had emerged in the field of severe respiratory viral infections. SARS-CoV-2 causes a wide spectrum of clinical symptoms from mild symptoms of upper respiratory tract infection to life-threatening pneumonia and adult respiratory distress syndrome (ARDS) [1,2]. Only patients with severe, and above all, critical stages of COVID-19 requiring oxygenation support, at least at the level of High-Flow Oxygen Therapy (HFOT), are admitted to intensive care. According to the up-to-date World Health Organization (WHO) clinical guidelines, patients in a critical stage of the disease suffer from ARDS (more detailed classification according to the Berlin criteria), and/or sepsis, and/or multiorgan dysfunction [3,4].

The incidence of severe and critical patients among all cases of COVID-19 is reported to be around 14% and 5–6%, respectively [5,6,7]. A considerable proportion of patients require intensive care unit (ICU) admission, with the literature indicating a range of 5% to 32% of hospitalized patients, and a significant percentage (ranging from 29% to 90%) necessitating invasive ventilation [5,8,9,10,11,12,13]. The critical stage of COVID-19 is associated with a high mortality rate [5]. However, there is ongoing debate regarding the occurrence and impact of bacterial pneumonia as a complication in patients with a critical stage of COVID-19 [14]. Unfortunately, evidence is scarce regarding the effect of concomitant bacterial pneumonia in this specific patient cohort.

Bacterial pneumonia in patients with COVID-19 can be defined as either co-infection, i.e., the recovery of bacterial respiratory pathogens in patients with COVID-19 at the time of a SARS-CoV-2 infection diagnosis, or *superinfection*, i.e., the subsequent acquisition of bacterial respiratory pathogens during the course of care for patients infected with SARS-CoV-2. Another classification of bacterial pneumonia in hospitalized COVID-19 patients is *community-acquired pneumonia* (CAP) and *hospital-acquired*/*nosocomial pneumonia* (HAP). In the literature, the term *co-infection* is often associated with CAP, and the term *superinfection* is associated with HAP [15]. In fact, a lot of patients came to the hospital about a week after the SARS-CoV-2 infection diagnosis for deterioration of their condition, i.e., in that case, diagnosed CAP should be classified as superinfection. In addition, the terms *co-* and *superinfection* also refer to non-lung infections and are actually used in the literature for various infections (urinary tract infection, wound infection, catheter-related bloodstream infection, *Clostridioides difficile* infections, etc.). This is why the above-mentioned terms should not be confused. In the present study, only patients with bacterial CAP and HAP are assessed.

The most frequent etiological agents expected to cause CAP and HAP in the context of the Czech Republic are listed in Table 1.

The incidence of bacterial co- and superinfections in COVID-19 patients are generally reported as low, at 3–4% and 5–16%, respectively [17,18,19,20]. As stated in the COVID-19 Treatment Guidelines and other articles, the incidence rate of all associated infections varies based on the disease severity, the duration of hospitalization, the diagnostic techniques utilized, and the timeframe of the study [1,21,22].

The fact that the incidences of bacterial respiratory co-infections and superinfections increase significantly with the severity of the disease is confirmed by other authors [17,21,22,23,24,25,26,27]. In patients requiring ICU care, the incidence of bacterial respiratory co-infection or CAP is reported to be 14–41%, and the incidence of bacterial lung superinfection or HAP ranges between 14–40% [24,27,28,29,30,31,32,33,34]. What is more, in patients with severe or critical stages of COVID-19, the risk for poor outcomes also increases significantly [15,21,22,29]. Many articles lack clear specifications regarding the types of infections (whether all infections or specifically pulmonary infections) that are included and analyzed in their studies [15].

Diagnosing bacterial CAP and HAP in patients affected by COVID-19 is challenging, as the symptoms of viral and bacterial pneumonia overlap significantly [7,35,36,37,38]. The traditional diagnostic criteria of CAP (clinical symptoms, radiological findings, general and laboratory signs of infection/inflammation, and microbiological findings) in patients with hypoxemic failure (and/or ARDS) due to COVID-19 pneumonia have numerous drawbacks [39]. During clinical examination, a more pronounced auscultation finding, the production of purulent sputum, and, sometimes, pleural pain may indicate an accompanying bacterial etiology [40,41]. Although the radiological findings in critical COVID-19 have a typical image, the picture develops over time, depends on the severity of the disease, and is highly variable [42,43]. Moreover, findings culminate 9–13 days after infection, i.e., at the time when bacterial HAP can be expected [44,45]. All of the above reduces the specificity and ability of radiological methods to distinguish bacterial superinfection reliably. The use of biochemical markers also has major limitations. COVID-19 is often initially presented with a high level of inflammatory markers, especially C-reactive protein (CRP), thus masquerading as a bacterial infection [46,47,48]. Even procalcitonin (PCT), which has been extensively studied, has been found to be unreliable in distinguishing between viral and bacterial etiology [49]. The reasons are that PCT levels can be elevated in several other serious conditions, including viral infections, lung aspiration, and renal failure, and due to the inability to establish a definitive threshold that can accurately differentiate viral pneumonia from bacterial pneumonia. [25,49,50,51,52,53,54]. Despite this fact, we noted a rise in the use of PCT testing to guide antimicrobial prescribing [55]. It seems that it is more important to monitor the dynamics of biochemical markers (typically after the decreasing trend, they increase again in incipient and developing HAP), rather than their absolute values [56]. Moreover, the traditional microbiological diagnostic tests for CAP and HAP, i.e., the cultivation of sputum and blood cultures, can fail to reveal a definitive pathogen [25,57].

This prospective observational monocentric cohort study was conducted with the objective of assessing the incidence of bacterial CAP and HAP, as well as their impact on mortality in a cohort of critically ill COVID-19 patients admitted to the intensive care unit (ICU) at University Hospital Olomouc between 1 November 2020 and 31 December 2022. The secondary objectives of this study were to identify the bacterial etiology of CAP and HAP and to explore the capabilities of diagnostic tools, with a focus on inflammatory biomarkers.

## 2. Results

**Figure 1:**
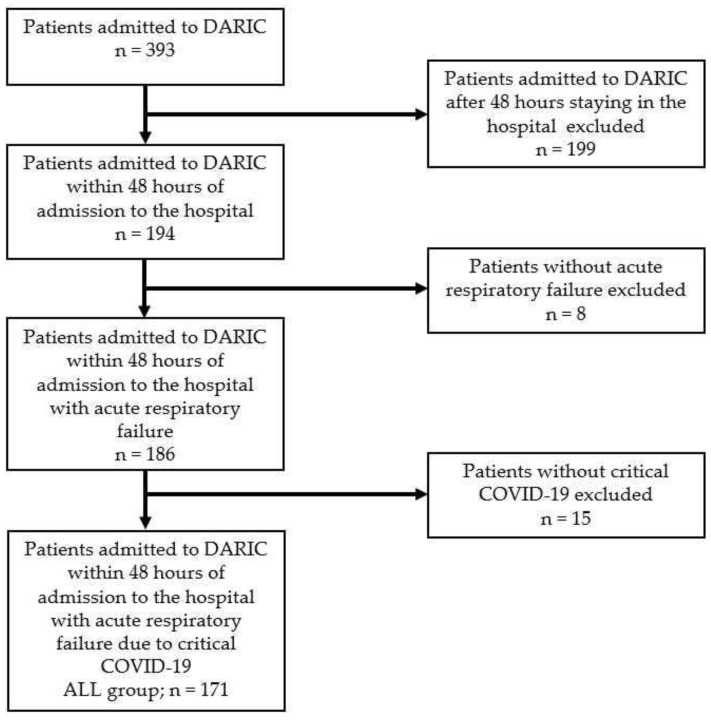
Flow chart demonstrating patient selection criteria.

Over the study period between 1 November 2020 and 31 December 2022, a total of 393 patients stayed at the ICU of the Department of Anaesthesiology, Resuscitation and Intensive Care (DARIC). After applying inclusion and exclusion criteria, the final study group (ALL group) comprised 171 patients (Figure 1).

The complete demographic data of the ALL group are shown in Table 2. The mean age in the ALL group was 62.9 (SD ± 12.3) years. The chi-squared test shows male gender predomination throughout the ALL group; the proportion of males was 64.3% (*p* = 0.002). The mean Acute physiology and chronic health evaluation score (APACHE) II on admission was 13.9 (SD ± 8.5), with patients 65 and over having a higher mean value (15.4 points) compared to patients under 65 years (12.4 points; *p* = 0.005). The mean Body Mass Index (BMI) was 33.3 (SD ± 7.2), with 65.0% of patients with a BMI over 30. Increased BMI had no association with a higher APACHE II score (*p* = 0.218). Invasive ventilation and HFOT were delivered to 69.0% and 86.5% of patients, respectively. The average Length of Stay (LOS) was 13 days (SD ± 5.2). Overall mortality reached 44.4%, of which 55.3% of patients died in palliative care.

While 86.5% of patients were treated by corticosteroids, no patients were given anti- interleukin-6 (anti-IL-6) or anti- interleukin-1 (anti-IL-1) treatment. A total of 25.1% of patients underwent treatment with remdesivir. The mortality in the age group under 65 years was 33.7%, while in the age group 65 and over, it was 54.5% (*p* = 0.009). There was no statistical difference in mortality between genders (Fisher’s exact test *p* = 0.785).

**Figure 2:**
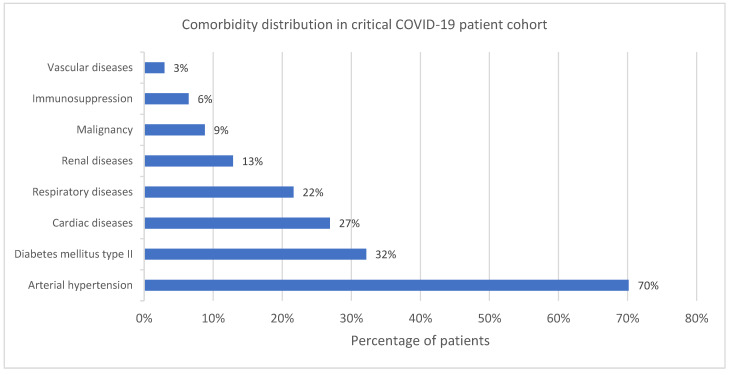
Comorbidity distribution in critical COVID-19 patient cohort.

**Figure 3:**
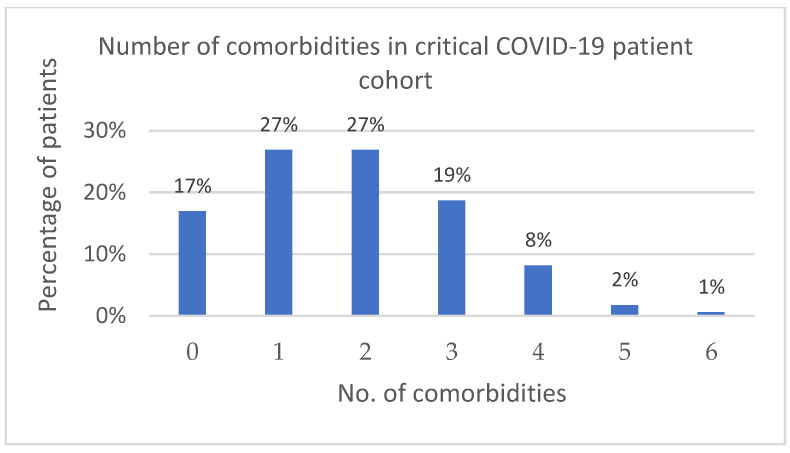
Number of comorbidities in critical COVID-19 patient cohort.

The comorbidities of the ALL group are summarized in Figure 2. The most common (70.2%) one was hypertension. A total of 83.0% of the patients suffered from at least 1 comorbidity (Figure 3).

### 2.1. Classification of ALL Group Patients according to Presence of Infections

**Figure 4:**
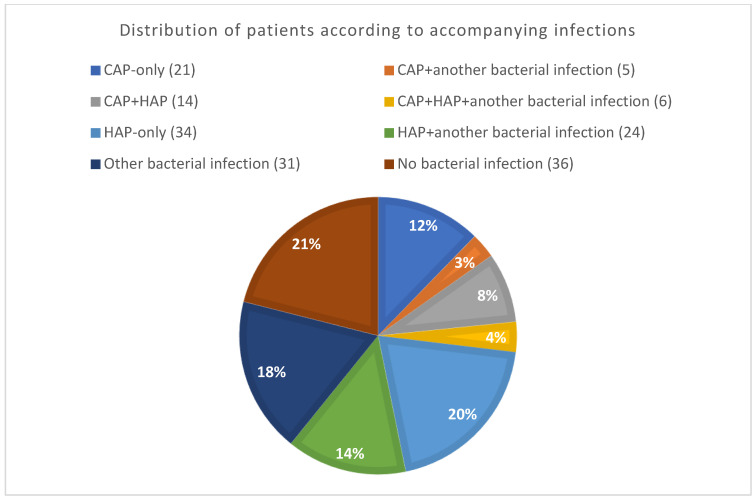
Distribution of patients according to accompanying infections (in brackets absolute number of patients).

Patients were classified into groups according to the presence of infections (bacterial HAP, bacterial CAP, bacterial HAP only, bacterial CAP only, and other bacterial infections). There were also patients who suffered from multiple bacterial infections, such as CAP and HAP, or pneumonia along with another bacterial infection. Lastly, there was also a group of patients without any bacterial infection during hospitalization. The group of these patients was designated as the No Bacterial Infectiongroup. Patients in this cohort experienced critical COVID-19 but did not develop any bacterial infection. The proportion of bacterial CAP, HAP, and other infections is shown in Figure 4.

### 2.2. Bacterial CAP, HAP, and Other Infection Groups

Of the total number of 171 patients, 46 (26.9%) suffered from CAP (the CAP group) upon admission, of whom 18 (39.1%) fulfilled the criteria of sepsis. Some of them developed HAP or other infections later. Therefore, the CAP-only group comprised 21 (12.3%) patients.

There were 78 (45.6%) patients who developed HAP (HAP group) during their stay at the DARIC. Of these patients, 34 (19.8%) patients developed HAP only without any other bacterial infection (the HAP-only group). Furthermore, 67 (39.2%) patients had no HAP or CAP. In addition, 36 (21.1%) patients showed no bacterial infection during their stay at the DARIC (No Bacterial Infection group). Moreover, 31 (18.1%) patients suffered from infections other than respiratory infections (urinary tract infection, wound infection, catheter-related bloodstream infection, *Clostridioides difficile* infections, etc.).

### 2.3. Critical COVID-19 Patients with Bacterial CAP

Of the total number of 171 patients, 46 (26.9%) had evidence of bacterial CAP upon admission. Of these 46 episodes, 27 (58.7%) were of monomicrobial etiology, 11 (23.9%) were of polymicrobial etiology, and 8 (17.4%) were without detection of bacterial pathogens. The most common etiologic agents were the following: *Chlamydophila pneumoniae* and *Mycoplasma pneumoniae* (22 patients, 47.8%); *Klebsiella pneumoniae/variicola*, all ESBL-positive (7, 15.2%); *Staphylococcus aureus* (5, 10.9%), of which 1 was a methicillin-resistant strain (MRSA); *Burkholderia multivorans* (4, 8.7%); *Enterobacter cloacae* (3, 6.5%) and *Escherichia coli* (2, 4.3%), both ESBL-positive; and then 1 strain (2.1%) each of *Proteus mirabilis*, ESBL-positive *Serratia marcescens*, *Pseudomonas aeruginosa*, *Acinetobacter baumannii*, *Achromobacter* sp., and *Moraxella catarrhalis* a *Haemophilus influenzae*.

Of these 46 CAP patients, 21 (45,7%) suffered only from bacterial CAP and no other infection but critical COVID-19 (the CAP-only group). The CAP-only group showed no statistical difference in gender, age, BMI, APACHE II, or respiratory support compared to the No Bacterial Infection group. The proportion of males was 70.5%. The most common comorbidity was hypertension, reaching 82.6% (the highest proportion from all analyzed groups), and the difference was statistically significant compared to the No Bacterial Infectiongroup at 61.1% (*p* = 0.005). CAP-only patients suffered mostly from one (32.6%) or two (21.7%) comorbidities. While LOS did not differ between the groups, mortality was significantly higher in the CAP-only group compared to the No Bacterial Infection group at 38.1% vs. 11.1% (*p* = 0.022; OR = 4.923; [95% CI] 1.261–19.227). A comparison of demography and characteristics is shown in Table 3.

### 2.4. Initial Inflammatory Markers in Bacterial CAP-Only Patients

Table 4 shows the values of inflammatory markers upon admission day in the CAP-only and No Bacterial Infection groups. There was a statistically significant difference in CRP (*p* = 0.014), PCT (*p* < 0.0001), IL-6 (*p* = 0.007), and WBC (*p* = 0.024), but not in temperature (*p* = 0.216), between the CAP-only and No Bacterial Infection groups.

The generated ROC curve of the initial values of inflammatory markers shows only good discriminatory abilities of the tests, with the best for PCT (AUC = 0.818) (Supplementary File S1). The optimal cut-off values to predict bacterial co-infection according to Youden’s J statistics in the studied cohort are CRP = 220 mg/L (SE = 0.524; SP = 0.806), PCT = 0.8 µg/L (SE = 0.667; SP = 0.944), and IL-6 = 40 ng/L (SE = 0.714; SP = 0.750).

### 2.5. Critical COVID-19 Patients with Bacterial HAP

Out of 171 patients, 78 (45.6%) developed HAP during their hospitalization at DARIC. The etiology was monobacterial in 51 patients (65.4%) and polybacterial in 27 (34.6%). The most frequent etiologic agents causing HAP were strains of *Klebsiella pneumoniae* (50 patients, 64.1%), *Burkholderia multivorans* (15 patients, 19.2%), *Pseudomonas aeruginosa* (12 patients, 15.4%), *Serratia marcescens* (10 patients, 12.8%), *Enterococcus faecium* (7 patients, 9.0%), *Enterobacter cloacae* (6 patients, 7.7%), *Stenotrophomonas maltophilia* (3 patients, 3.8%), and *Escherichia coli* (2 patients, 2.6%), followed by 1 strain (1.3%) each of *Proteus mirabilis*, *Morganella morganii*, and *Staphylococcus aureus*.

**Figure 5:**
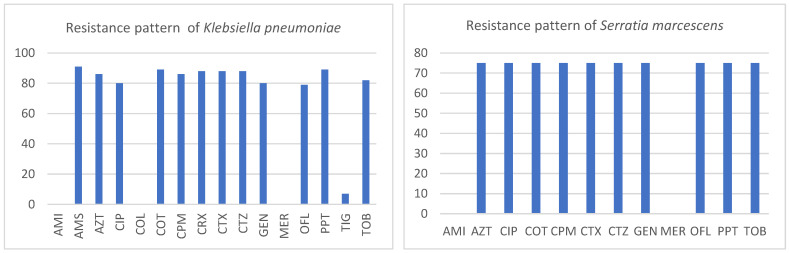

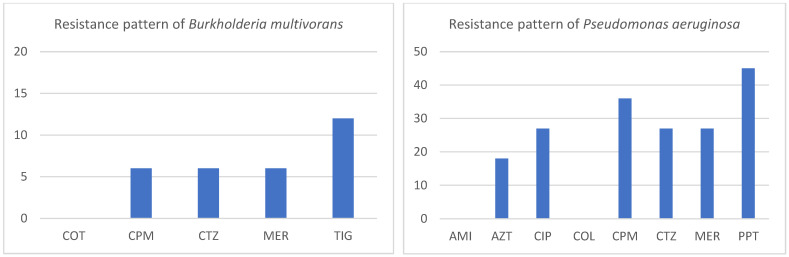
Resistance pattern of the most frequent species causing HAP (in %). Legend: AMI—amikacin, AMS—ampicillin/sulbactam, AZT—aztreonam, CIP—ciprofloxacin, COL—colistin, COT—co-trimoxazole, CPM—cefepime, CRX—cefuroxime, CTX—cefotaxime, CTZ—ceftazidime, GEN—gentamicin, MER—meropenem, OFL—ofloxacin, PPT—piperacillin/tazobactam, TIG—tigecycline, TOB—tobramycin.

Resistance to antibiotics in the most common species is shown in Figure 5. The vast majority of *Klebsiella pneumoniae* strains (more than 80.0%) were ESBL producers and multi-resistant (resistant to antibiotics from three or more antibiotic groups). They retain good susceptibility only to meropenem, tigecycline, amikacin, and colistin. Also, *Serratia marcescens* strains were more than 70.0% multi-resistant, with retained susceptibility only to meropenem and amikacin. Out of the 10 strains of *Serratia marcescens*, only 2 phenotypes were found: one with 3 strains susceptible to all tested antibiotics and the other one with 7 multidrug-resistant strains showing the same antibiogram (susceptible only to meropenem and amikacin).

The resistance rate of *Pseudomonas aeruginosa* to meropenem was almost 30.0%, as well as to ceftazidime and ciprofloxacin. Resistance to cefepime and piperacillin/tazobactam was even higher. Strains of *Burkholderia multivorans* showed low resistance to broad-spectrum beta-lactams except for aztreonam.

**Figure 6:**
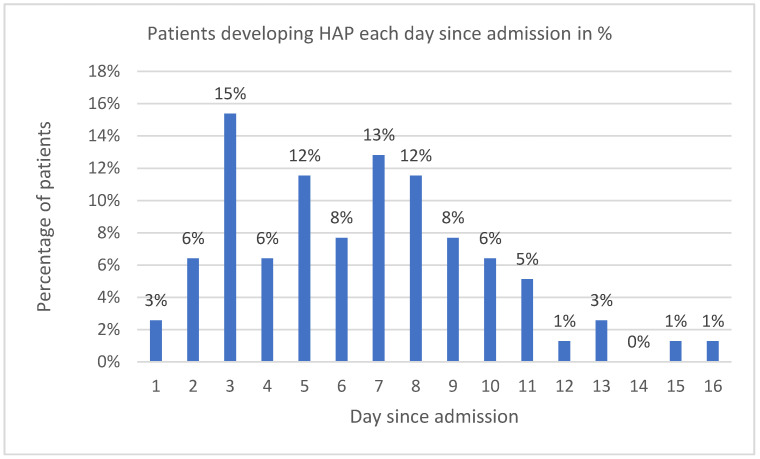
Patients developing HAP each day since admission in %.

Of the 78 HAP patients, 34 (43.6%) suffered only from HAP (HAP-only group) and no other infection. The HAP-only group showed no statistical difference in gender, age, BMI, APACHE II, and HFOT compared to the No Bacterial Infection group. Invasive ventilation was more frequent in the HAP-only group, showing a statistical difference compared to the No Bacterial Infection group at 73.5% vs. 47.2% (*p* = 0.03). The proportion of males was higher in the HAP-only group than in the No Bacterial Infection group, at 80.2% vs. 63.9%, but not statistically significant (*p* = 0.109) (Table 5). From the most common comorbidities, hypertension was represented in 79.4% of HAP-only patients compared to 61.1% in the No Bacterial Infection group (*p* = 0.121). Most of the HAP-only patients suffered from one to three comorbidities (mean 1.97, SD ± 1.09), with the last being the most frequent (26.9%). Bacterial HAP developed on day 8.2 on average. Figure 6 shows the percentage of patients who developed HAP every day since admission. LOS and mortality were significantly higher compared to patients in the No Bacterial Infection group. LOS in HAP-only patients was 15.6 days to 9.56 days (*p* < 0.0001). Mortality in HAP-only patients reached 55.9% vs. 11.1% in the No Bacterial Infection group (*p* < 0.0001; OR = 10.133; 95% CI, 2.931–35.032).

### 2.6. Inflammatory Markers in Bacterial HAP-Only Patients

The inflammatory markers in the HAP-only group and the No Bacterial Infection group upon admission to the hospital were compared, and we found statistically significant differences in the values of PCT and IL-6 but not in other markers listed below (Table 6).

The ROC curve was generated for the initial values of PCT and IL-6 markers, and it showed poor discriminatory abilities to predict HAP (Supplementary File S2). For PCT, the optimal cut-off was difficult to find due to the low sensitivity with increasing specificity. For IL-6, the optimal cut-off of initial values to predict bacterial superinfection was 40 ng/L (SE = 0.667; SP = 0.750) according to Youden’s J statistic.

The peak values of the inflammatory markers in the HAP-only group (when suspicion of secondary bacterial pneumonia arose) were assessed and compared with the average values of the inflammatory markers in the No Bacterial Infection group. We found statistically significant differences in CRP, PCT, IL-6, and WBC. The difference in body temperature was also significant. The complete results are given in Supplementary File S3.

Then, an ROC analysis of significant results was performed (Supplementary File S4), and based on this, the best predictor of HAP in critical COVID-19 patients appeared to be IL-6, with an AUC = 0.810. The optimal cut-off value of IL-6 determining HAP was 65 ng/L (SE = 0.818 and SP = 0.686). The optimal cut-off value for the change (dynamics) in IL-6 determining HAP increased by 33 ng/L (SE = 0.667 and SP 0.829). The optimal cut-off value for CRP was 165 mg/L, and for PCT, it was 0.3 µg/L.

### 2.7. Signs and Symptoms of HAP

Sputum production, respiratory and circulatory function deterioration, and positive X-ray findings were evaluated. In the HAP-only group, 52.9% of patients produced sputum compared to 2.8% in the No Bacterial Infection group, 91.2% experienced a deterioration in respiratory functions vs. 11.1%, 61.8% vs.16.7% showed worsening of circulatory functions, and 45.2% vs. 7.1% had a new positive X-ray finding typical of bacterial pneumonia. Multivariate logistic regression was performed in order to find the best independent predictor of HAP (Table 7). The best independent predictor was respiratory function deterioration, with an OR of 20.45 (95% CI, 6.866–60.929).

## 3. Material and Methods

### 3.1. Study Design

This project was conducted as a prospective observational monocentric cohort study. The data of all patients in the critical stage of COVID-19 pneumonia admitted to the ICU of the Department of Anaesthesiology, Resuscitation and Intensive Care (DARIC), University Hospital Olomouc and Faculty of Medicine and Dentistry, Palacky University Olomouc, were prospectively collected between 1 November 2020 and 31 December 2022. Informed consent for the presentation of anonymized data was obtained from the patients or their legal representatives upon admission to the hospital. This study was approved by the Institutional Ethics Committee with reference number 213/21. Patient data were assessed by ICU physicians and clinical microbiologists.

### 3.2. Setting

The University Hospital Olomouc (UHO) is one of the largest healthcare facilities in the Czech Republic (1200 beds), providing medical care to approximately 925,000 outpatients and 50,000 inpatients per year. The highest level of intensive care is provided by the DARIC. The department admits critically ill individuals with any diagnoses except primary cardiac surgery patients.

In the spring of 2020, the DARIC was completely transformed into an ICU for critical COVID-19 patients. During the main pandemic waves, between the autumn of 2020 and the autumn of 2021, the capacity of the department was extended repeatedly to as many as 35 beds (350%), mostly (approximately 70%) in the open-space arrangement, divided into four or five halls. A total of 97% of patients suffered from ARDS and/or sepsis, which is critical COVID-19, as defined by WHO [4].

### 3.3. Study Group of Patients

The study included patients over 18 years of age who met the WHO criteria of critical COVID-19 (positive SARS-CoV-2 PCR test by either nasopharyngeal swab or endotracheal aspirate, X-ray or CT scan showing bilateral opacities, lobar or pulmonary collapse, or nodules, acute respiratory failure requiring HFOT therapy (min. flow 50 L/min, FiO_2_ 50%) or invasive ventilation and PaO_2_/FiO_2_ less than or equal to 200 mm Hg) and were admitted to DARIC within 48 h of hospitalization. In our study, no patients on non-invasive ventilation were included because this technique was not used during the pandemic waves. Patients, who were admitted to DARIC after 48 h of hospitalization in UHO or other hospitals or for other reasons (for example, major surgery, stroke, trauma) were excluded.

At the latest upon their admission to the department, some COVID-19 patients were started on intensive oxygen therapy (HFOT). Other patients were already intubated upon their transfer from lower-level facilities or an emergency department or following their resuscitation for hypoxic cardiac arrest. A considerable proportion of patients had to be intubated within hours or days after admission for respiratory function deterioration despite HFOT. The condition of some invasively ventilated patients worsened so that they met the criteria for extracorporeal membrane oxygenation (ECMO) initiation.

### 3.4. Definition of Bacterial CAP and HAP

CAP was defined as bacterial pneumonia diagnosed concurrently with SARS-CoV-2 infection, or within less than 48 hours of hospital admission.

HAP was defined as bacterial pneumonia diagnosed in patients hospitalized with SARS-CoV-2 infection on the third day of hospitalization or later.

### 3.5. Diagnostic Criteria for Bacterial CAP and HAP

Diagnosis of bacterial CAP and HAP was based on clinical, radiological, and microbiological findings (e.g., more pronounced auscultation, purulent or hemorrhagic sputum production and/or pleural pain, radiological evidence of consolidations consistent with bacterial pneumonia, positive/negative sputum cultivation, detection of etiological agents of atypical pneumonia).

Bacterial CAP was confirmed if other sources/sites of bacterial infection were excluded and at least three of the four following criteria were positive:

- Positive detection of bacterial pathogens;
- PCT ≥ 1.0 µg/L, or CRP ≥ 100 mg/L;
- Clinical signs of bacterial CAP (sputum production, auscultation);
- Consolidations in lung tissue consistent with bacterial pneumonia shown by X-ray or CT scan;

Bacterial HAP was confirmed if the following criteria were fulfilled:

- Dynamics in inflammatory biomarkers: if a new peak occurred after the initial decline (at least three of the following: PCT, CRP, IL-6, and white blood cell count (WBC)). Thresholds have been set CRP ≥ 50 mg/L, PCT ≥ 0.5 µg/L, IL-6 ≥ 300 ng/L or WBC ≥ 11 × 10^9^;
- Positive detection of bacterial pathogens.
- And at least three of the six following criteria:
  - Deterioration of clinical condition
  - Respiratory insufficiency progression (increase in FiO_2_, PEEP, pressure support, need for intubation)
  - New sputum production (change in amount/colour)
  - Fever (new onset)
  - Circulation instability (decrease in mean arterial pressure ≤ 65 torr, onset or increase in vasopressor support)
  - New infiltrate on lung X-ray or CT scan

Only the first episode of nosocomial pneumonia was evaluated.

### 3.6. Data Collected during the Data Collection Phase

- Age;
- Gender;
- BMI;
- APACHE II;
- LOS;
- Mortality on day 28 (D28);
- Palliative care;
- Comorbidities;
- Biomarkers (CRP, PCT, IL-6, WBC);
- Body temperature;
- Clinical signs of sepsis;
- Microbiological findings.

### 3.7. Microbiological Examination

COVID-19 was diagnosed by direct virus nucleic acid detection by RT-PCR identifying three specific gene areas of viral RNA in nasopharyngeal and/or oropharyngeal swabs [58]. Clinical samples from the upper or lower airways (nasopharyngeal swabs or sputum in non-intubated patients, airway secretions in intubated patients), urine, and stool were collected regularly (on admission and then twice a week) and cultivated in all patients as a screening of microbial colonization. When clinical suspicions of infectious complications arose, relevant samples were also taken at that moment. Samples were processed and evaluated using standard microbiological procedures.

The relevant microbiological methods for the detection of etiological agents expected to cause CAP and HAP were used as follows:

- Cultivation and microscopy of the clinical sample from the lower respiratory tract;
- Blood cultivation;
- Direct serological detection of pneumococcal and legionella antigens from urine;
- PCR detection of bacterial nucleic acid;
- Serological method for detection of antibodies against mycoplasma and chlamydophila.

Bacterial pathogens were identified using the MALDI-TOF MS system (Biotyper Microflex, Bruker Daltonics, Bremen, Germany). Susceptibility to antibiotics was determined with a standard microdilution method in accordance with the EUCAST criteria [59]. To ensure quality control, the following reference bacterial strains were used: *Escherichia coli* ATCC 25922, *Pseudomonas aeruginosa* ATCC 27853, *Staphylococcus aureus* ATCC 29213, and *Enterococcus faecalis* ATCC 29212.

The production of Extended Spectrum Beta-Lactamases (ESBLs) and AmpC beta-lactamases and carbapenemases was detected by phenotypic tests and confirmed by PCR detection of relevant genes [60,61,62,63,64]. All *Staphylococcus aureus* strains were tested for resistance to methicillin using selective diagnostic chromogenic media (Colorex/TM/MRSA, TRIOS, Prague, Czech Republic) and an immunochromatographic assay for the detection of PBP2a (PBP2a SA Culture Colony Test, Alere^TM^, Abbott, Prague, Czech Republic). Positive results were confirmed by the detection of the *mecA* gene [65]. Vancomycin resistance in enterococci was confirmed by the detection of the *vanA* and *vanB* genes [66]. Multi-drug resistance, defined as the resistance of the bacterial isolate to antibiotics of three or more antibiotic classes, was evaluated in the isolated bacteria.

Serological diagnostics of chlamydophila antibodies (IgA, IgM, and IgG) and mycoplasma antibodies (IgM and IgG) were performed by a quantitative assay by immunoluminometric method, utilizing a LIAISON^®^XL analyzer (DiaSorin, Prague, Czech Republic). Pneumococcal and legionella antigens were directly detected from urine by immunochromatographic methods (BIOSYNEX^®^ S. pneumoniae (Biosynex, Illkirch-Graffenstaden, France) and BinaxNOW—Legionella Urinary Antigen Card (Abbott, Prague, Czech Republic)). Direct detection of the nucleic acid of atypical bacteria (*Chlamydophila pneumoniae*, *Mycoplasma pneumoniae*, *Legionella pneumophila*, and *Bordetella pertussis* and *parapertussis*) was carried out by real-time PCR (Allplex™ PneumoBacter Assay, Seegen, Seoul, Republic of Korea).

### 3.8. Statistical Analysis

Continuous variables were reported as medians and standard deviations (SDs), and categorical variables were expressed as counts and percentages.

Between-group differences in baseline characteristics were assessed using Fisher’s exact test for qualitative variables and the Mann–Whitney *U* test for quantitative variables. All tests were performed at the 0.05 significance level.

A receiver operating characteristic (ROC) curve and the area under the curve (AUC) were generated to try to determine the predictive and optimal cut-off values of inflammatory markers of bacterial CAP and HAP in critical COVID-19 patients. The optimal cut-off values were determined based on Younden’s index, which maximizes the sum of the sensitivity and specificity.

Multiple logistic regression analysis was used to identify the risk factor of bacterial HAP in patients with critical COVID-19 pneumonia. For the independent predictors of HAP, the main clinical signs and symptoms of pneumonia, which were assessed in daily practice, were chosen.

The distribution of genders in the studied cohort was compared by the chi-squared goodness-of-fit test.

The statistical software IBM SPSS Statistics version 23.0 for Windows (IBM, Armonk, NY, USA) was used for statistical processing.

## 4. Discussion

Based on the findings, the incidence of bacterial CAP and HAP in the cohort of critical COVID-19 patients was high at 27% and 46%, respectively.

These figures present a significant discrepancy compared to certain studies, which report values of 3–5% and 5–18% for all hospitalized patients and 8% and 40% in the ICU setting [17,18,19,20]. Still, the range of the values is quite wide throughout the different sources. Also, there are inconsistencies in the definitions of co-infection and superinfection, or definitions are missing [15]. Our patient population is very homogeneous in this respect—only patients who were admitted to ICU within 48 hours since their admission to the hospital (without any previous stay in another health care facility) were evaluated, so it can be argued that respiratory infection, which was diagnosed only at the moment of admission to ICU, was indeed CAP. Any other lung infection (after the second day of admission to the ICU) was labeled as HAP. 

The variations in incidence across the literature are very likely attributable to the heterogeneity of the patient population, demographics studied (the severity of COVID-19, age, and associated diseases), access to care, regional differences, and infection prevention and control measures implemented. This idea is supported by multiple authors [18,67]. Numerous studies have included a diverse patient population, with a relatively small proportion of critically ill individuals. Logically, therefore, they describe low incidences of accompanying bacterial respiratory infections [68]. According to the literature, the critical stage of COVID-19 affects 5-6% of patients, while the severe form affects approximately 14% [5,6,7]. Studies exclusively dealing with patients requiring care at ICU have already described higher incidences: 14–36% for CAP and 8–42% for HAP [27,36,68,69]. 

The higher incidence of bacterial CAP and HAP in our patient cohort can be primarily attributed to the severe condition of patients due to the critical stage of COVID-19, the high prevalence of comorbidities within our group, and the older age of our patients.

The Department of Anaesthesiology, Resuscitation and Intensive Care, University Hospital Olomouc, exclusively admitted the most severe cases from the hospital as well as the entire catchment area. All patients enrolled in this study had PaO_2_ /FiO_2_ levels below 200 mmHg, with the majority below 150 mmHg. It is important to note that our patient cohort is homogeneous, unlike other studies that fail to mention the severity status of their patients, such as APACHE or SOFA scores upon admission, the COVID-19 stage, and/or hypoxemic index. This uniformity allows us to draw more specific conclusions regarding the incidence of CAP and HAP in critically ill COVID-19 patients. Based on the existing literature, it is evident that the incidence of concurrent infections increases as the severity of COVID-19 increases [21,22,23,24,25,26,27]. This could be the main reason for the high incidence of bacterial CAP and HAP in our dataset. 

Considering the fact that previous studies did not find an increase in healthcare-associated infections (HAIs) among non-COVID-19 patients during the pandemic, there is a hypothesis that CAP and HAP could be related to the pathophysiology of COVID-19 [70]. Indeed, the literature describes a causal relationship between viral respiratory tract infections and the development of bacterial infections [71]. The extent of the incidence will undoubtedly be influenced by the damaged respiratory tract epithelium, as well as other pathophysiological and immunological mechanisms. However, environmental issues also play a significant role. Previous studies by our authors’ team, utilizing genetic methods, demonstrated clonal spread of identical bacterial strains in the ICU during the pandemic, leading to exogenous nosocomial infections, including respiratory tract infections caused by *Klebsiella pneumoniae*, *Serratia marcescens*, and *Burkholderia cepacia* complex, as well as colitis caused by *Clostridioides difficile* [72,73]. The reasons for this can be attributed to the predominantly open-space layout of the ward, where most rooms are open-plan, with few enclosed spaces for the isolation of patients, many of whom were connected to an HFOT device via a nasal mask. This device delivered a high flow of fresh gases and allowed for direct exhalation into the surrounding environment. The potential for spreading infected aerosols through this route has been a topic of extensive discussion in the literature [74,75,76,77,78]. Furthermore, it is plausible that during the initial months of the pandemic, when the DARIC department underwent restructuring, the influx of beds, staff, and patients played a significant role in the dissemination of bacteria within the ICU [72,73]. Hygienic epidemiological regimes were effective against SARS-CoV-2 infections in healthcare workers but were not sufficiently effective against the clonal spread of bacterial strains. 

The possible influence of corticosteroids, IL-6 inhibitors, antivirals, and vaccination on the incidence rates of CAP and HAP is worth considering. However, the potential correlation of these medications and vaccination with the occurrence of bacterial infections in our patient cohort has not been investigated due to the reasons outlined below. The administration of corticosteroids varied significantly among patients during the study period, reflecting the existing evidence and evolution of guidelines related to the type, dosage, and indication of these drugs over time. This variability prevented us from conducting a statistical analysis to determine the true effect of corticosteroids on patient outcomes. While the current literature does not indicate an increased risk of bacterial infections with corticosteroid therapy, it is crucial to assess an individual patient’s risk and immune response profile, as hypo- and normo-inflammatory patients may not benefit from corticosteroid treatment and could even experience increased mortality [79,80,81]. IL-6 inhibitors were only approved by the Food and Drug Administration in December 2022 for the treatment of COVID-19, after our study had already concluded. Remdesivir was administered to only 25.1% of our patient cohort, largely due to delays in hospital admission that resulted in failure to meet the drug’s indication criteria. Regarding vaccination, we lack specific data for our cohort of patients, as the critical COVID-19 group primarily consisted of unvaccinated individuals who developed severe/critical illness. Vaccinated individuals typically only experienced mild to moderate symptoms and were, therefore, not included in the study. Exceptions included patients with hematological malignancies, organ transplant recipients, and severe immunodeficiencies. Thus, the majority of our patient population during the later stages of the pandemic comprised older, unvaccinated individuals. All these associations mentioned above (the severity of COVID-19, age, comorbidities, delay in hospital admission, and the low rate of vaccination in the study cohort) could potentially contribute to the high mortality rate observed in patients admitted to the ICU with critical COVID-19. In our examined cohort of patients, the cumulative mortality rate on the 28th day (D28) was recorded at 44%. Considering the severity of the disease and the high rate of associated infections, this number is favorable compared to other sources. According to a meta-analysis by Armstrong et al., it reached 42% in May 2020 and decreased slightly to 36% later [82]. It varied significantly in different regions of the world and throughout time as new methods, drugs, and vaccinations were introduced. Another work by De Santis cites data that associated mortality in patients requiring intensive care, ranging between 19% and 62% [17]. 

Co- and superinfections in patients with COVID-19 pneumonia are associated with the severity of the disease and poor outcomes [15,17,31,83]. Notably, in the subgroup of patients with CAP as the only bacterial accompanying infection, the mortality rate on D28 reached 38% (OR, 4.9), which is five times more compared to the group without any bacterial infection. Furthermore, among patients with HAP alone, the mortality rate on D28 was even more pronounced, reaching 56% (OR, 10.1), ten times more compared to the group without any bacterial infection.

These data are quite unique, as sources regarding mortality in patients with critical COVID-19 and bacterial pulmonary superinfections are scarce and inconclusive. There are studies that encompass all stages of COVID-19 and various types of superinfections, including UTIs, catheter-related sepsis, and others. Some studies have suggested a higher mortality rate among patients with bacterial superinfections, while others have not confirmed this association [31,84,85,86]. Other types of studies have consistently shown a distinct pattern, indicating a higher prevalence of bacterial infections among non-survivors compared to survivors [22,87,88].

The observation that bacterial pneumonia in COVID-19 patients is linked to elevated mortality rates can be attributed to several factors associated with the underlying viral illness. These factors include a compromised pulmonary microbiome, impaired localized immune defenses, and the presence of a cytokine storm. These conditions collectively contribute to the severity of ARDS and the unfavorable prognosis associated with secondary infections in critically ill patients. Moreover, bacterial pneumonia exacerbates pre-existing conditions, thereby magnifying the risk of mortality.

Unfortunately, a dearth of suitable data for comparative analysis in relation to the study outcomes was encountered during the comprehensive review of the available literature. Particularly, data on the mortality rates of critically ill COVID-19 patients with bacterial pneumonia compared to a group of critical COVID-19 patients without bacterial respiratory superinfection were not found.

It should be emphasized that an assessment of the presence of bacterial CAP or HAP in patients with severe/critical COVID-19 courses is complicated [32,33,35,36,56]. The traditional criteria (clinical signs, radiological findings, and general and laboratory signs of infection/inflammation) are burdensome to apply to patients experiencing hypoxemic failure and/or ARDS. Specifically, interpreting radiological findings becomes challenging due to ground glass opacities (GGOs) and consolidations with a bilateral and peripheral distribution, which are characteristic features of COVID-19 pneumonia [44]. Clinical criteria, such as auscultation findings and sputum production, generally lack sensitivity and specificity in the intensive care setting, especially among patients requiring mechanical ventilation. The significance of inflammatory markers remains uncertain, and distinguishing between bacterial infection and colonization based on microbiological examinations, particularly tracheal aspirates, poses difficulties.

The role of inflammatory biomarkers in diagnosing bacterial pneumonia in patients with critical COVID-19 remains obscure. Several factors can influence the levels of inflammatory parameters, including the hyperinflammatory response, the virulence of microbial agents, COVID-19-induced immunosuppression, the administration of corticosteroids, IL-6 inhibitors such as tocilizumab, etc. [89]. The role of different phenotypes of COVID-19-associated ARDS (CARDS), hyperinflammatory versus hypo/normo-inflammatory, is being discussed too [81]. In our studied cohort, inflammatory markers and clinical signs showed statistical differences between critical COVID-19 patients with bacterial pneumonia and those without any bacterial infection. The best predictive value for CAP was found with PCT (AUC, 0.818). The best sensitivity and specificity are shown with a cut-off value of 0.8 µg/L. As demonstrated, e.g., in a study by van Berkel et al., the presence of bacterial pneumonia in COVID-19 is usually associated with higher PCT levels [56]. According to Berkel’s study, the presence of a secondary bacterial infection can be confirmed by PCT levels exceeding 1.0 ng/L, while levels below 0.25 ng/L effectively exclude such an infection [56]. Pink et al. demonstrated a 94% negative predictive value for identifying secondary bacterial infection in COVID-19 patients with PCT values below 0.55 µg/L [53]. Harte et al. observed median PCT values well below this threshold both in the infection and no-infection groups [55]. Within our examined cohort, the discriminatory capabilities of PCT for HAP were found to be uncertain. Despite the notably elevated initial levels of PCT in patients with HAP compared to the bacterial-infection-free cohort, this biomarker exhibits limited predictive capability for superinfection, as the identification of a reasonable cut-off value remains elusive. These observations may be attributed to the administration of corticosteroids and COVID-19-induced immunosuppression [89]. Certain authors argue that PCT values have proven inadequate in confirming bacterial infection, whereas others contend that our understanding in this area remains inconclusive [55,90]. A significant emphasis in the literature is placed on the correlation between elevated PCT levels and a severe disease course and/or fatal outcomes [91].

Our analysis of peak values for inflammatory biomarkers in HAP patients showed that even though there was a significant difference in all measured inflammatory markers, only IL-6 had a good predictive value for HAP (AUC 0.81), with an optimal cut-off value of 65 ng/L, and a rise by at least 33 ng/L. Nevertheless, the literature data have indicated that proinflammatory cytokines, such as IL-6, play a pivotal role in the development of acute lung injury in COVID-19 [92]. Consequently, some clinicians prefer using IL-6 inhibitors, such as tocilizumab, to mitigate lung damage in COVID-19 patients. However, the potential risk of developing bacterial HAP in patients treated with tocilizumab remains uncertain [93] as well as its use in the hypoinflammatory phenotype of CARDS. In their research, Liu et al. assert that serum levels of IL-6 and CRP can effectively evaluate disease severity and predict patient outcomes in COVID-19 cases [94]. Thus, it is plausible to also consider IL-6 as a prognostic factor for disease severity.

Regarding the clinical signs of pneumonia, the multiple logistic regression model applied in our studied patient cohort identified respiratory deterioration as the most significant independent predictor, with an OR of 20.5. Notably, this indicator was present in 91% of patients with HAP. Ideally, the diagnosis would be supported by the presence of sputum production or other typical signs of bacterial pneumonia, such as pleuritic pain, as well as the detection of proven bacterial agents in biological samples through direct methods such as culture or PCR, or indirect methods such as the identification of antibodies in serum. However, it is important to note that the etiological agent may not always be cultivable, especially if antibiotic therapy has already begun. Additionally, it is important to proceed with caution when interpreting radiological findings, as the imaging features of COVID-19 pneumonia may mimic those of various other infectious and non-infectious conditions [41,44].

In terms of the etiology of concomitant respiratory infections in patients with critical COVID-19, in this study, the most common etiologic agents in CAP were chlamydophila and mycoplasmas, Enterobacterales (including 71% multidrug-resistant strains), Gram-negative non-fermenting rods (*Pseudomonas aeruginosa*, *Acinetobacter baumannii*, *Achromobacter* sp.), and *Staphylococcus aureus*, with 20% MRSA. In HAP, the etiological agents were mainly Enterobacterales and Gram-negative non-fermenting rods, with a high resistance rate in Enterobacterales. 

Studies investigating the etiology of bacterial pneumonia in patients with critical COVID-19 predominantly identify community-associated pathogens (such as *Streptococcus pneumoniae*, *Haemophilus influenzae*, *Staphylococcus aureus*, and *Moraxella catarrhalis*) in CAP. These pathogens have also been detected in hospital-acquired pneumonia (HAP) cases [19,95,96,97]. Contrary to these results, in our study, bacterial species typically associated with nosocomial infections have been identified as causative agents of CAP (alongside chlamydophila and mycoplasma). This may be due to the high severity of the clinical condition of critical COVID-19 patients, who may have been colonized easily and quickly by hospital pathogens. The spread of multi-drug resistant clones in the hospital environment that has been adapted to the dramatic wave of a viral pandemic is also likely to play a role [73]. This conjecture is supported by the results of microbiological findings in HAP patients with a high prevalence of multi-drug-resistant Enterobacterales strains. Interestingly, for example, in *Serratia marcescens*, only two phenotypes were found: one with three strains sensitive to all tested antibiotics and the second one included seven strains with the same antibiogram (sensitive only to meropenem and amikacin). This implies the importance of knowing the current and local epidemiological situation in a given department, as the prevalence of bacterial species can be distorted by local outbreaks. A multi-center study conducted in four ICUs in the Czech Republic in 2013-2015 (DARIC was one of the four ICUs) revealed that the most frequent etiologic species of HAP were *Klebsiella pneumoniae, Pseudomonas aeruginosa*, *Escherichia coli*, *Enterobacter* spp., *Staphylococcus aureus*, and *Burkholderia cepacia* complex [98]. As shown, the frequency of pathogens causing HAP in critical COVID-19 patients is quite different than it was 7 years ago in the nationwide study. This highlights the requirement for microbiological laboratories to provide up-to-date overviews of the frequency of bacterial species from specific biological materials and their resistance rates.

## 5. Limitations

Our study is subject to several limitations that should be acknowledged. Firstly, despite being a prospective study, it is important to note that it is conducted at a single center, leading to potential biases associated with a monocentric design.

Additionally, although the initial enrolment aimed to include a larger number of patients, we ultimately encountered a limited number of participants in the specific groups under comparison. Consequently, generalizability and statistical power may be impacted by the small cohort sizes.

Nevertheless, it is worth highlighting that our study population, as well as individual subgroups, were precisely defined, ensuring clarity in the characterization of patients with CAP and HAP based on multiple established criteria.

However, it is important to acknowledge that our study did not investigate the potential influence of yeast or viral infections, which could potentially act as confounding factors. This warrants consideration when interpreting the results and their implications.

In conclusion, while our study has notable strengths in terms of clear population and subgroup definitions, it is crucial to bear in mind the limitations inherent in its monocentric design, the limited number of participants in certain groups, and the absence of investigation into yeast or viral infections other than COVID-19.

## 6. Conclusions

The incidence of CAP and HAP in patients with critical COVID-19 in the intensive care unit was 27% and 46%, respectively. Critical COVID-19 patients suffering from bacterial CAP and HAP showed higher mortality rates compared to patients without any bacterial infection: 38% and 56% vs. 11%. Critical COVID-19 patients suffering from bacterial HAP had a longer LOS compared to patients without any infection (15.6 days vs. 9.6 days).

Our study revealed that the most prevalent causative agents of CAP were chlamydophila and mycoplasmas, followed by Enterobacterales (with 71% of strains displaying multidrug resistance), Gram-negative non-fermenting rods (including *Pseudomonas aeruginosa*, *Acinetobacter baumannii*, and *Achromobacter* sp.), and *Staphylococcus aureus*, of which 20% were methicillin-resistant (MRSA). Regarding HAP, Enterobacterales and Gram-negative non-fermenting rods were the frequent etiological agents, with a notable presence of multidrug-resistant strains among Enterobacterales.

The diagnosis of concurrent bacterial pneumonia in critically ill COVID-19 patients remains a complex task. Our findings suggest that for CAP diagnosis, it is advisable to evaluate the initial levels of inflammatory markers, preferably PCT, alongside typical clinical manifestations indicative of bacterial infection and/or positive microbiological results. In the case of HAP diagnosis, it is crucial to conduct daily thorough clinical assessments, particularly focusing on changes in respiratory function, while closely monitoring the dynamics of inflammatory markers, preferably IL-6.

## Figures and Tables

**Table 1:** The most frequent etiological agents expected to cause CAP and HAP [16].

CAP	HAP
*Streptococcus pneumoniae*	*Klebsiella pneumoniae*
*Haemophilus influenzae*	*Pseudomonas aeruginosa*
*Mycoplasma pneumoniae*	*Escherichia coli* and other Enterobacterales
*Chlamydophila pneumoniae*	*Staphylococcus aureus*
*Chlamydophila psittaci*	*Burkholderia cepacia* complex
*Moraxella catarrhalis*	*Acinetobacter baumannii*
*Staphylococcus aureus*	*Stenotrophomonas maltophilia*
*Legionella pneumophila*	
*Bordetella pertussis* and *parapertussis*	

**Table 2:** Demographic data of the studied cohort.

Variables	ALL Group (*n* = 171)	HAP Group (*n* = 78)	CAP Group (*n* = 46)	No Bacterial Infection Group (*n* = 36)
Age, mean (SD)	62.9 (±12.3)	62.6 (±11.5)	63.3 (±13.6)	64 (±13.23)
Male, *n* (%)	110 (64.3)	55 (70.5)	33 (71.7)	23 (63.9)
BMI, kg/m^2^, mean (SD)	33.3 (±7.2)	32.8 (±7.7)	33 (±7.3)	32.5 (±5.7)
LOS (ICU), mean (SD)	13 (±5.2)	15.6 (±4.9)	13.1 (±6)	9.7 (±3.4)
Mortality (D28), *n* (%)	76 (44.4)	51 (65.4)	27 (58.7)	4 (11.1)
Palliative care, *n* (%)	42 (24.6)	28 (35.9)	20 (43.5)	1 (2.8)
Mech. ventilation, *n* (%)	118 (69.0)	66 (84.6)	35 (76.1)	17 (47.2)
HFOT, *n* (%)	148 (86.5)	66 (84.6)	36 (78.3)	32 (88.9)
APACHE II, mean (SD)	13.9 (±8.5)	13.9 (±7.8)	17.4 (±10.4)	12.7 (±7.55)
Corticosteroids, *n* (%)	148 (86.5)	68 (87.2)	42 (91.3)	32 (88.9)

Comparison of patients in our cohort. Values are numbers or means ± standard deviations. The dataset was analyzed by the Mann–Whitney *U* test or Fisher’s exact test, as appropriate (*p* < 0.005).

**Table 3:** Demographics and characteristics of CAP-only group and No Bacterial Infection group.

Variables	CAP-Only (*n* = 21)	No Bacterial Infection (*n* = 36)	*p*-Value
Age, mean (SD)	63.3 (±13.44)	64 (±13.23)	0.709
Male, *n* (%)	15 (71.4)	23 (63.9)	0.772
BMI, kg/m^2^, mean (SD)	33.6 (±7.53)	32.5 (±5.7)	0.785
LOS (ICU), mean (SD)	11.6 (±6.1)	9.7 (±3.4)	0.231
Mortality (D28), *n* (%)	8 (38.1)	4 (11.1)	0.022
Palliative care, *n* (%)	5 (23.8)	1 (2.8)	0.022
Mech. ventilation, *n* (%)	13 (61.9)	17 (47.2)	0.41
HFOT, *n* (%)	19 (90.5)	32 (88.9)	1.0
APACHE II, mean (SD)	17.1 (±11.82)	12.7 (±7.55)	0.177
Corticosteroids	19 (90.5)	32 (88.9)	1.0

**Table 4:** Inflammatory markers in CAP-only and No Bacterial Infection groups upon admission.

Variables	CAP-Only (*n* = 21)	No Bacterial Infection (*n* = 36)	*p*-Value
CRP, mean (SD)	209 (±91.1)	148 (±78.2)	0.014
PCT, mean (SD)	7.1 (±21.8)	0.3 (±0.3)	<0.0001
IL-6, mean (SD)	1282.3 (±5122.7)	57.7 (±129.2)	0.007
WBC × 10^9^, mean (SD)	12.4 (±6)	9.3 (±3.7)	0.024
Temperature, mean (SD)	37.1 (±1.1)	36.9 (±0.9)	0.216

**Table 5:** Demographics and characteristics of the HAP-only group and the No Bacterial Infection group.

Variables	HAP-Only (*n* = 34)	No Bacterial Infections (*n* = 36)	*p*-Value
Age, mean (SD)	64.6 (±10.4)	64 (±13.23)	0.558
Male, *n* (%)	28 (82.4)	23 (63.9)	0.109
BMI, kg/m^2^, mean (SD)	32.5 (±5.7)	32.5 (±5.7)	0.698
LOS (ICU), mean (SD)	15.6 (±5.0)	9.7 (±3.4)	0.227
Mortality (D28), *n* (%)	19 (55.9)	4 (11.1)	<0.0001
Palliative care, *n* (%)	9 (26.5)	1 (2.8)	0.006
Mech. ventilation, *n* (%)	25 (73.5)	17 (47.2)	0.03
HFOT, *n* (%)	31 (91.2)	32 (88.9)	1.0
APACHE II, mean (SD)	14.2 (±7.7)	12.7 (±7.55)	0.393
Corticosteroids	27 (79.4)	32 (88.9)	0.336

**Table 6:** Initial inflammatory markers in the HAP-only and No Bacterial Infection groups.

Variables Initial Values	HAP-Only (*n* = 34)	No Bacterial Infections (*n* = 36)	*p*-Value
CRP, mean (SD)	140.5 (±65.3)	148 (±78.2)	0.716
PCT, mean (SD)	1.3 (±2.9)	0.3 (±0.3)	0.032
IL-6, mean (SD)	192.7 (±639.4)	57.7 (±129.2)	0.005
WBC × 10^9^, mean (SD)	8.19 (±9.9)	9.3 (±3.7)	0.145
Temperature, mean (SD)	37.2 (±1.2)	36.9 (±0.9)	0.285

**Table 7:** Predictors of HAP evaluated with multivariate logistic regression.

	OR	95% CI for OR		Sig.
		Lower	Upper	
Sputum (1)	3.629	1.122	11.737	0.31
Vasopressor (1)	1.924	0.689	5.371	0.212
Respiratory deterioration (1)	20.453	6.866	60.929	<0.0001
X-ray (1)	6.435	1.959	21.133	0.002

## Data Availability

The datasets analyzed during this study are available from the corresponding author upon reasonable request.

## Supplementary Materials

The following supporting information can be downloaded at: https://www.mdpi.com/article/10.3390/antibiotics13020192/s1, Supplementary File S1: The ability of inflammatory markers to predict CAP analysis. Supplementary File S2: The ability of the initial values of PCT and IL-6 to predict HAP analysis. Supplementary File S3: Peak inflammatory markers in HAP-only and the No Bacterial Infection groups. Supplementary File S4: The ability of the peak values of PCT and IL-6 and their changes to predict HAP.

## References

[1] Coronavirus Disease 2019 (COVID-19) Treatment Guidelines. https://covid19treatmentguidelines.nih.gov.

[2] Chen N., Zhou M., Dong X., Qu J., Gong F., Han Y., Qiu Y., Wang J., Liu Y., Wei Y. (2020). Epidemiological and clinical characteristics of 99 cases of 2019 novel coronavirus pneumonia in Wuhan, China: A descriptive study. Lancet.

[3] Ranieri V.M., Rubenfeld G.D., Thompson B.T., Ferguson N.D., Caldwell E., Fan E., Camporota L., Slutsky A.S., ARDS Definition Task Force (2012). Acute respiratory distress syndrome: The Berlin Definition. JAMA.

[4] World Health Organization (WHO) Clinical Management of COVID-19: Living Guideline. https://www.who.int/publications/i/item/WHO-2019-nCoV-clinical-2023.2.

[5] Wu Z., McGoogan J.M. (2020). Characteristics of and Important Lessons from the Coronavirus Disease 2019 (COVID-19) Outbreak in China: Summary of a Report of 72,314 Cases from the Chinese Center for Disease Control and Prevention. JAMA.

[6] Hajjar L.A., Costa I.B.S.D.S., Rizk S.I., Biselli B., Gomes B.R., Bittar C.S., de Oliveira G.Q., de Almeida J.P., de Oliveira Bello M.V., Garzillo C. (2021). Intensive care management of patients with COVID-19: A practical approach. Ann. Intensive Care.

[7] Phua J., Weng L., Ling L., Egi M., Lim C.M., Divatia J.V., Shrestha B.R., Arabi Y.M., Ng J., Gomersall C.D. (2020). Intensive care management of coronavirus disease 2019 (COVID-19): Challenges and recommendations. Lancet Respir. Med..

[8] Grasselli G., Pesenti A., Cecconi M. (2020). Critical Care Utilization for the COVID-19 Outbreak in Lombardy, Italy: Early Experience and Forecast During an Emergency Response. JAMA.

[9] Abate S.M., Ahmed A.S., Mantfardo B., Basu B. (2020). Rate of Intensive Care Unit admission and outcomes among patients with coronavirus: A systematic review and Meta-analysis. PLoS ONE.

[10] Guan W.J., Ni Z.Y., Hu Y., Liang W.H., Ou C.Q., He J.X., Liu L., Shan H., Lei C.L., Hui D.S.C. (2020). Clinical Characteristics of Coronavirus Disease 2019 in China. N. Engl. J. Med..

[11] Wunsch H. (2020). Mechanical Ventilation in COVID-19: Interpreting the Current Epidemiology. Am. J. Respir. Crit. Care Med..

[12] Johnson J.A., Mallari K.F., Pepe V.M., Treacy T., McDonough G., Khaing P., McGrath C., George B.J., Yoo E.J. (2023). Mechanically ventilated COVID-19 patients admitted to the intensive care unit in the United States with or without respiratory failure secondary to COVID-19 pneumonia: A retrospective comparison of characteristics and outcomes. Acute Crit. Care.

[13] Ouyang L., Yu M., Zhu Y., Gong J. (2021). Respiratory Supports Of COVID-19 Patients in Intensive Care Unit: A Systematic Review. Heliyon.

[14] Galli F., Bindo F., Motos A., Fernández-Barat L., Barbeta E., Gabarrús A., Ceccato A., Bermejo-Martin J.F., Ferrer R., Riera J. (2023). Procalcitonin And C-Reactive Protein to Rule Out Early Bacterial Coinfection In COVID-19 Critically Ill Patients. Intensive Care Med..

[15] Feldman C., Anderson R. (2021). The role of co-infections and secondary infections in patients with COVID-19. Pneumonia.

[16] Kolář M., Rejman D., Bardoň J. (2020). Zásady Antibiotické Léčby [Principles of Antibiotic Treatment].

[17] De Santis V., Corona A., Vitale D., Nencini C., Potalivo A., Prete A., Zani G., Malfatto A., Tritapepe L., Taddei S. (2022). Bacterial infections in critically ill patients with SARS-2-COVID-19 infection: Results of a prospective observational multicenter study. Infection.

[18] Langford B.J., So M., Raybardhan S., Leung V., Westwood D., MacFadden D.R., Soucy J.R., Daneman N. (2020). Bacterial co-infection and secondary infection in patients with COVID-19: A living rapid review and meta-analysis. Clin. Microbiol. Infect..

[19] Garcia-Vidal C., Sanjuan G., Moreno-García E., Puerta-Alcalde P., Garcia-Pouton N., Chumbita M., Fernandez-Pittol M., Pitart C., Inciarte A., Bodro M. (2021). Incidence of co-infections and superinfections in hospitalized patients with COVID-19: A retrospective cohort study. Clin. Microbiol. Infect..

[20] Hughes S., Troise O., Donaldson H., Mughal N., Moore L.S.P. (2020). Bacterial and fungal coinfection among hospitalized patients with COVID-19: A retrospective cohort study in a UK secondary-care setting. Clin. Microbiol. Infect..

[21] Yang X., Yu Y., Xu J., Shu H., Xia J., Liu H., Wu Y., Zhang L., Yu Z., Fang M. (2020). Clinical course and outcomes of critically ill patients with SARS-CoV-2 pneumonia in Wuhan, China: A single-centered, retrospective, observational study. Lancet Respir. Med..

[22] Zhou F., Yu T., Du R., Fan G., Liu Y., Liu Z., Xiang J., Wang Y., Song B., Gu X. (2020). Clinical course and risk factors for mortality of adult inpatients with COVID-19 in Wuhan, China: A retrospective cohort study. Lancet.

[23] Zhang G., Hu C., Luo L., Fang F., Chen Y., Li J., Peng Z., Pan H. (2020). Clinical features and short-term outcomes of 221 patients with COVID-19 in Wuhan, China. J. Clin. Virol..

[24] Fattorini L., Creti R., Palma C., Pantosti A., Unit of Antibiotic Resistance and Special Pathogens, The Unit of Antibiotic Resistance and Special Pathogens (2020). Bacterial coinfections in COVID-19: An underestimated adversary. Ann. Dell’Istituto Super. Sanita.

[25] Metlay J.P., Waterer G.W. (2020). Treatment of Community-Acquired Pneumonia During the Coronavirus Disease 2019 (COVID-19) Pandemic. Ann. Intern. Med..

[26] Elabbadi A., Turpin M., Gerotziafas G.T., Teulier M., Voiriot G., Fartoukh M. (2021). Bacterial coinfection in critically ill COVID-19 patients with severe pneumonia. Infection.

[27] Lansbury L., Lim B., Baskaran V., Lim W.S. (2020). Co-infections in people with COVID-19: A systematic review and meta-analysis. J. Infect..

[28] Verroken A., Scohy A., Gérard L., Wittebole X., Collienne C., Laterre P.F. (2020). Co-infections in COVID-19 critically ill and antibiotic management: A prospective cohort analysis. Crit. Care.

[29] Youngs J., Wyncoll D., Hopkins P., Arnold A., Ball J., Bicanic T. (2020). Improving antibiotic stewardship in COVID-19: Bacterial co-infection is less common than with influenza. J. Infect..

[30] Clancy C.J., Nguyen M.H. (2020). Coronavirus Disease 2019, Superinfections, and Antimicrobial Development: What Can We Expect?. Clin. Infect. Dis..

[31] Yoon S.M., Lee J., Lee S.M., Lee H.Y. (2023). Incidence and clinical outcomes of bacterial superinfections in critically ill patients with COVID-19. Front. Med..

[32] De Francesco M.A., Signorini L., Piva S., Pellizzeri S., Fumarola B., Corbellini S., Piccinelli G., Simonetti F., Carta V., Mangeri L. (2023). Bacterial and fungal superinfections are detected at higher frequency in critically ill patients affected by SARS CoV-2 infection than negative patients and are associated to a worse outcome. J. Med. Virol..

[33] Schouten J., De Waele J., Lanckohr C., Koulenti D., Haddad N., Rizk N., Sjövall F., Kanj S.S., Alliance for the Prudent Use of Antibiotics (APUA) (2021). Antimicrobial stewardship in the ICU in COVID-19 times: The known unknowns. Int. J. Antimicrob. Agents.

[34] Gao C.A., Markov N.S., Stoeger T., Pawlowski A., Kang M., Nannapaneni P., Grant R.A., Pickens C., Walter J.M., Kruser J.M. (2023). Machine Learning Links Unresolving Secondary Pneumonia to Mortality in Patients with Severe Pneumonia, Including COVID-19. J. Clin. Investig..

[35] Rawson T.M., Moore L.S.P., Zhu N., Ranganathan N., Skolimowska K., Gilchrist M., Satta G., Cooke G., Holmes A. (2020). Bacterial and Fungal Coinfection in Individuals with Coronavirus: A Rapid Review to Support COVID-19 Antimicrobial Prescribing. Clin. Infect. Dis..

[36] Wu H.Y., Chang P.H., Chen K.Y., Lin I.F., Hsih W.H., Tsai W.L., Chen J.A., Lee S.S., GREAT working group (2022). Coronavirus disease 2019 (COVID-19) associated bacterial coinfection: Incidence, diagnosis and treatment. J. Microbiol. Immunol. Infect..

[37] Mandell L.A., Zhanel G.G., Rotstein C., Muscedere J., Loeb M., Johnstone J. (2022). Community-Acquired Pneumonia in Canada During Coronavirus Disease 2019. Open Forum Infect. Dis..

[38] Torres A., Niederman M.S., Chastre J., Ewig S., Fernandez-Vandellos P., Hanberger H., Kollef M., Li Bassi G., Luna C.M., Martin-Loeches I. (2017). International ERS/ESICM/ESCMID/ALAT guidelines for the management of hospital-acquired pneumonia and ventilator-associated pneumonia: Guidelines for the management of hospital-acquired pneumonia (HAP)/ventilator-associated pneumonia (VAP) of the European Respiratory Society (ERS), European Society of Intensive Care Medicine (ESICM), European Society of Clinical Microbiology and Infectious Diseases (ESCMID) and Asociación Latinoamericana del Tórax (ALAT). Eur. Respir. J..

[39] Heneghan C., Pludermann A., Mahtani K. Differentiating Viral from Bacterial Pneumonia. https://www.cebm.net/covid-19/differentiating-viral-from-bacterial-pneumonia.

[40] COVID-19 Rapid Guideline: Managing: Managing COVID-19. https://www.nice.org.uk/guidance/ng191.

[41] Naranje P., Bhalla A.S., Jana M., Garg M., Nair A.D., Singh S.K., Banday I. (2022). Imaging of Pulmonary Superinfections and Co-Infections in COVID-19. Curr. Probl. Diagn. Radiol..

[42] Altmayer S., Zanon M., Pacini G.S., Watte G., Barros M.C., Mohammed T.L., Verma N., Marchiori E., Hochhegger B. (2020). Comparison of the computed tomography findings in COVID-19 and other viral pneumonia in immunocompetent adults: A systematic review and meta-analysis. Eur. Radiol..

[43] Parekh M., Donuru A., Balasubramanya R., Kapur S. (2020). Review of the Chest CT Differential Diagnosis of Ground-Glass Opacities in the COVID Era. Radiology.

[44] Duzgun S.A., Durhan G., Demirkazik F.B., Akpinar M.G., Ariyurek O.M. (2020). COVID-19 pneumonia: The great radiological mimicker. Insights Imaging.

[45] Zeng F., Huang Y., Guo Y., Yin M., Chen X., Xiao L., Deng G. (2020). Association of inflammatory markers with the severity of COVID-19: A meta-analysis. Int. J. Infect. Dis..

[46] Luan Y.Y., Yin C.H., Yao Y.M. (2021). Update Advances on C-Reactive Protein in COVID-19 and Other Viral Infections. Front. Immunol..

[47] Sidhwani S.K., Mirza T., Khatoon A., Shaikh F., Khan R., Shaikh O.A., Nashwan A.J. (2023). Inflammatory markers and COVID-19 disease progression. J. Infect. Public Health.

[48] Wunderink R.G., Waterer G. (2017). Advances in the causes and management of community acquired pneumonia in adults. BMJ.

[49] Kamat I.S., Ramachandran V., Eswaran H., Guffey D., Musher D.M. (2020). Procalcitonin to Distinguish Viral from Bacterial Pneumonia: A Systematic Review and Meta-Analysis. Clin. Infect. Dis..

[50] Sabahat U., Thomas L.M., Shaikh N.A., Ali N.M. (2023). An Unexpected Cause of Raised Procalcitonin. Dubai Med. J..

[51] Cuquemelle E., Soulis F., Villers D., Roche-Campo F., Ara Somohano C., Fartoukh M., Kouatchet A., Mourvillier B., Dellamonica J., Picard W. (2011). Can procalcitonin help identify associated bacterial infection in patients with severe influenza pneumonia? A multicentre study. Intensive Care Med..

[52] Pfister R., Kochanek M., Leygeber T., Brun-Buisson C., Cuquemelle E., Machado M.B., Piacentini E., Hammond N.E., Ingram P.R., Michels G. (2014). Procalcitonin for diagnosis of bacterial pneumonia in critically ill patients during 2009 H1N1 influenza pandemic: A prospective cohort study, systematic review and individual patient data meta-analysis. Crit. Care.

[53] Pink I., Raupach D., Fuge J., Vonberg R.P., Hoeper M.M., Welte T., Rademacher J. (2021). C-reactive protein and procalcitonin for antimicrobial stewardship in COVID-19. Infection.

[54] May M., Chang M., Dietz D., Shoucri S., Laracy J., Sobieszczyk M.E., Uhlemann A.C., Zucker J., Kubin C.J. (2021). Limited Utility of Procalcitonin in Identifying Community-Associated Bacterial Infections in Patients Presenting with Coronavirus Disease 2019. Antimicrob. Agents Chemother..

[55] Harte E., Kumarasamysarma S., Phillips B., Mackay O., Rashid Z., Malikova N., Mukit A., Ramachandran S., Biju A., Brown K. (2023). Procalcitonin Values Fail to Track the Presence of Secondary Bacterial Infections in COVID-19 Icu Patients. Antibiotics.

[56] van Berkel M., Kox M., Frenzel T., Pickkers P., Schouten J., RCI-COVID-19 study group (2020). Biomarkers for antimicrobial stewardship: A reappraisal in COVID-19 times?. Crit. Care.

[57] Lidman C., Burman L.G., Lagergren A., Ortqvist A. (2002). Limited value of routine microbiological diagnostics in patients hospitalized for community-acquired pneumonia. Scand. J. Infect. Dis..

[58] Zhang W., Du R.H., Li B., Zheng X.S., Yang X.L., Hu B., Wang Y.Y., Xiao G.F., Yan B., Shi Z.L. (2020). Molecular and serological investigation of 2019-nCoV infected patients: Implication of multiple shedding routes. Emerg. Microbes Infect..

[59] European Committee on Antimicrobial Susceptibility Testing Breakpoint Tables for Interpretation of MICs and Zone Diameters. http://www.eucast.org/clinical_breakpoints.

[60] Htoutou Sedlakova M., Hanulik V., Chroma M., Hricova K., Kolar M., Latal T., Schaumann R., Rodloff A.C. (2011). Phenotypic detection of broad-spectrum beta-lactamases in microbiological practice. Med. Sci. Monit..

[61] Tamma P.D., Simner P.J. (2018). Phenotypic Detection of Carbapenemase-Producing Organisms from Clinical Isolates. J. Clin. Microbiol..

[62] Dallenne C., Da Costa A., Decré D., Favier C., Arlet G. (2010). Development of a set of multiplex PCR assays for the detection of genes encoding important beta-lactamases in Enterobacteriaceae. J. Antimicrob. Chemother..

[63] Mlynarcik P., Dolejska M., Vagnerova I., Kutilová I., Kolar M. (2021). Detection of clinically important β-lactamases by using PCR. FEMS Microbiol. Lett..

[64] Pérez-Pérez F.J., Hanson N.D. (2002). Detection of plasmid-mediated AmpC beta-lactamase genes in clinical isolates by using multiplex PCR. J. Clin. Microbiol..

[65] Oliveira D.C., de Lencastre H. (2002). Multiplex PCR strategy for rapid identification of structural types and variants of the mec element in methicillin-resistant *Staphylococcus aureus*. Antimicrob. Agents Chemother..

[66] Dutka-Malen S., Evers S., Courvalin P. (1995). Detection of glycopeptide resistance genotypes and identification to the species level of clinically relevant enterococci by PCR. J. Clin. Microbiol..

[67] Schoettler J.J., Sandrio S., Boesing C., Bauer L., Miethke T., Thiel M., Krebs J. (2023). Bacterial Co- or Superinfection in Patients Treated in Intensive Care Unit with COVID-19- and Influenza-Associated Pneumonia. Pathogens.

[68] Vaughn V.M., Gandhi T.N., Petty L.A., Patel P.K., Prescott H.C., Malani A.N., Ratz D., McLaughlin E., Chopra V., Flanders S.A. (2021). Empiric Antibacterial Therapy and Community-onset Bacterial Coinfection in Patients Hospitalized with Coronavirus Disease 2019 (COVID-19): A Multi-hospital Cohort Study. Clin. Infect. Dis..

[69] Ramanan M., Burrell A., Paul E., Trapani T., Broadley T., McGloughlin S., French C., Udy A. (2021). Nosocomial infections amongst critically ill COVID-19 patients in Australia. J. Clin. Virol. Plus.

[70] Bussolati E., Cultrera R., Quaranta A., Cricca V., Marangoni E., La Rosa R., Bertacchini S., Bellonzi A., Ragazzi R., Volta C.A. (2022). Effect of the Pandemic Outbreak on ICU-Associated Infections and Antibiotic Prescription Trends in Non-COVID19 Acute Respiratory Failure Patients. J. Clin. Med..

[71] Oliva J., Terrier O. (2021). Viral and Bacterial Co-Infections in The Lungs: Dangerous Liaisons. Viruses.

[72] Bogdanová K., Doubravská L., Vágnerová I., Hricová K., Pudová V., Röderová M., Papajk J., Uvízl R., Langová K., Kolář M. (2021). *Clostridioides difficile* and Vancomycin-Resistant Enterococci in COVID-19 Patients with Severe Pneumonia. Life.

[73] Doubravská L., Htoutou Sedláková M., Fišerová K., Pudová V., Urbánek K., Petrželová J., Röderová M., Langová K., Mezerová K., Kučová P. (2022). Bacterial Resistance to Antibiotics and Clonal Spread in COVID-19-Positive Patients on a Tertiary Hospital Intensive Care Unit, Czech Republic. Antibiotics.

[74] Ip M., Tang J.W., Hui D.S., Wong A.L.N., Chan M.T.V., Joynt G.M., So A.T.P., Hall S.D., Chan P.K.S., Sung J.J.Y. (2007). Airflow and droplet spreading around oxygen masks: A simulation model for infection control research. Am. J. Infect. Control.

[75] Leung C.C.H., Joynt G.M., Gomersall C.D., Wong W.T., Lee A., Ling L., Chan P.K.S., Lui P.C.W., Tsoi P.C.Y., Ling C.M. (2019). Comparison of high-flow nasal cannula versus oxygen face mask for environmental bacterial contamination in critically ill pneumonia patients: A randomized controlled crossover trial. J. Hosp. Infect..

[76] Kotoda M., Hishiyama S., Mitsui K., Tanikawa T., Morikawa S., Takamino A., Matsukawa T. (2020). Assessment of the potential for pathogen dispersal during high-flow nasal therapy. J. Hosp. Infect..

[77] Li J., Fink J.B., Ehrmann S. (2020). High-flow nasal cannula for COVID-19 patients: Low risk of bio-aerosol dispersion. Eur. Respir. J..

[78] Elshof J., Hebbink R., Duiverman M.L., Hagmeijer R. (2020). High-flow nasal cannula for COVID-19 patients: Risk of bio-aerosol dispersion. Eur. Respir. J..

[79] Johns M., George S., Taburyanskaya M., Poon Y.K. (2022). A Review of the Evidence for Corticosteroids in COVID-19. J. Pharm. Pract..

[80] Moreno-Torres V., de Mendoza C., de la Fuente S., Sánchez E., Martínez-Urbistondo M., Herráiz J., Gutiérrez A., Gutiérrez Á., Hernández C., Callejas A. (2022). Bacterial Infections in Patients Hospitalized with COVID-19. Intern. Emerg. Med..

[81] Sinha P., Furfaro D., Cummings M.J., Abrams D., Delucchi K., Maddali M.V., He J., Thompson A., Murn M., Fountain J. (2021). Latent Class Analysis Reveals COVID-19–Related Acute Respiratory Distress Syndrome Subgroups with Differential Responses to Corticosteroids. Am. J. Respir. Crit. Care Med..

[82] Armstrong R.A., Kane A.D., Kursumovic E., Oglesby F.C., Cook T.M. (2021). Mortality in patients admitted to intensive care with COVID-19: An updated systematic review and meta-analysis of observational studies. Anaesthesia.

[83] Musuuza J.S., Watson L., Parmasad V., Putman-Buehler N., Christensen L., Safdar N. (2021). Prevalence and outcomes of co-infection and superinfection with SARS-CoV-2 and other pathogens: A systematic review and meta-analysis. PLoS ONE.

[84] Iacovelli A., Oliva A., Siccardi G., Tramontano A., Pellegrino D., Mastroianni C.M., Venditti M., Palange P. (2023). Risk Factors and Effect on Mortality of Superinfections in A Newly Established COVID-19 Respiratory Sub-Intensive Care Unit at University Hospital in Rome. BMC Pulm. Med..

[85] Nassar Y., Mokhtar A., Elhadidy A., Elsayed M., Mostafa F., Rady A., Eladawy A., Elshazly M., Saeed M., Mokhtar S. (2021). Outcomes and Risk Factors for Death in Patients with Coronavirus Disease-2019 (COVID-19) Pneumonia Admitted to The Intensive Care Units of An Egyptian University Hospital. A Retrospective Cohort Study. J. Infect. Public Health.

[86] Pickens C.O., Gao C.A., Cuttica M.J., Smith S.B., Pesce L.L., Grant R.A., Kang M., Morales-Nebreda L., Bavishi A.A., Arnold J.M. (2021). Bacterial Superinfection Pneumonia in Patients Mechanically Ventilated For COVID-19 Pneumonia. Am. J. Respir. Crit. Care Med..

[87] Wang L., He W., Yu X., Hu D., Bao M., Liu H., Zhou J., Jiang H. (2020). Coronavirus Disease 2019 in Elderly Patients: Characteristics and Prognostic Factors Based On 4-Week Follow-Up. J. Infect..

[88] Ferrando C., Mellado-Artigas R., Gea A., Arruti E., Aldecoa C., Bordell A., Adalia R., Zattera L., Ramasco F., Monedero P. (2020). Características, Evolución Clínica Y Patient characteristics, clinical course and factors associat-ed to ICU mortality in critically ill patients infected with SARS-CoV-2 in Spain: A prospective, cohort, multicentre study. Rev Esp Anestesiol Reanim (Engl Ed). Rev. Española Anestesiol. Reanim..

[89] Michailides C., Paraskevas T., Karalis I., Koniari I., Pierrakos C., Karamouzos V., Marangos M., Velissaris D. (2023). Impact of Bacterial Infections On COVID-19 Patients: Is Timing Important?. Antibiotics.

[90] Heer R.S., Mandal A.K.J., Kho J., Szawarski P., Csabi P., Grenshaw D., Walker I.A.L., Missouris C.G. (2021). Elevated Procalcitonin Concentrations in Severe COVID-19 May Not Reflect Bacterial Co-Infection. Ann. Clin. Bio-Chem. Int. J. Lab. Med..

[91] Vazzana N., Dipaola F., Ognibene S. (2022). Procalcitonin and Secondary Bacterial Infections in Covid-19: Association with Disease Severity and Outcomes. Acta Clin. Belg..

[92] Osuchowski M.F., Winkler M.S., Skirecki T., Cajander S., Shankar-Hari M., Lachmann G., Monneret G., Venet F., Bauer M., Brunkhorst F.M. (2021). The COVID-19 Puzzle: Deciphering Pathophysiology and Phenotypes of a New Disease Entity. Lancet Respir. Med..

[93] Adre L.A.B., Catangui J.S., Bondoc M.K.V., Abdurahman K.M. (2023). Prevalence of Hospital-Acquired Pneumonia Among Patients with Severe to Critical COVID-19 Pneumonia Given Tocilizumab. Cureus.

[94] Liu F., Li L., Xu M.D., Wu J., Luo D., Zhu Y.S., Li B.X., Song X.Y., Zhou X. (2020). Prognostic Value of Interleukin-6, C-Reactive Protein, And Procalcitonin in Patients with COVID-19. J. Clin. Virol..

[95] Barrasa H., Martín A., Maynar J., Rello J., Fernández-Torres M., Aguirre-Quiñonero A., Canut-Blasco A., Alava COVID-19 Study Investigators (2021). High rate of infections during ICU admission of patients with severe SARS-CoV-2 pneumonia: A matter of time?. J. Infect..

[96] Zhu X., Ge Y., Wu T., Zhao K., Chen Y., Wu B., Zhu F., Zhu B., Cui L. (2020). Co-infection with respiratory pathogens among COVID-19 cases. Virus Res..

[97] Contou D., Claudinon A., Pajot O., Micaëlo M., Longuet Flandre P., Dubert M., Cally R., Logre E., Fraissé M., Mentec H. (2020). Bacterial and viral co-infections in patients with severe SARS-CoV-2 pneumonia admitted to a French ICU. Ann. Intensive Care.

[98] Herkel T., Uvizl R., Doubravska L., Adamus M., Gabrhelik T., Htoutou Sedlakova M., Kolar M., Hanulik V., Pudova V., Langova K. (2016). Epidemiology of hospital-acquired pneumonia: Results of a Central European multicenter, prospective, observational study compared with data from the European region. Biomed. Pap..

