# Supplementary material for: Bacterial Community- and Hospital-Acquired Pneumonia in Patients with Critical COVID-19—A Prospective Monocentric Cohort Study
Source: Antibiotics (Basel). 2024 Feb 16;13(2):192. doi: 10.3390/antibiotics13020192 (PMC10886267; doi:10.3390/antibiotics13020192)
Supplement: Supplementary file 1 [file antibiotics-13-00192-s001.zip › antibiotics-2764843-supplementary.pdf]

Supplement 1. The ability of inflammatory markers to predict CAP analysis.

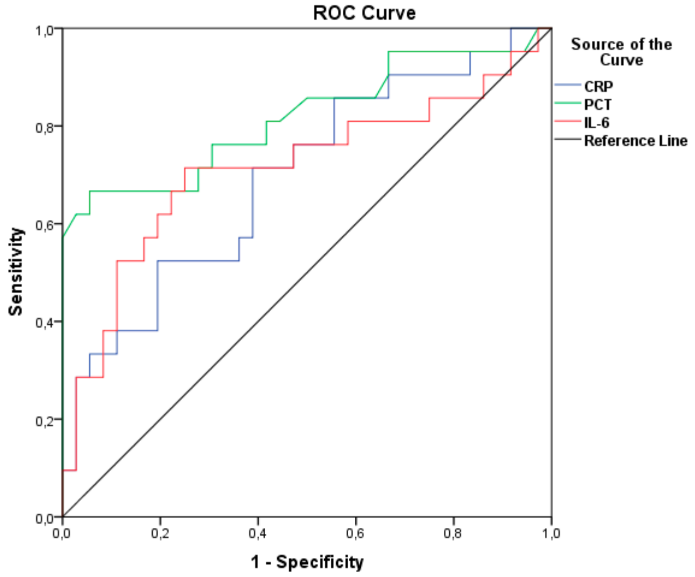

Area Under the Curve

Test Result Variable(s)	Area	p[b]	Asymptotic 95% Confidence Interval	
			Lower Bound	Upper Bound
CRP	0.696	0.014	0.552	0.839
PCT	0.818	0.0001	0.690	0.946
IL-6	0.714	0.007	0.562	0.867

b. Null hypothesis: true area = 0.5

Supplement 2. The ability of initial values of PCT and IL-6 to predict HAP analysis.

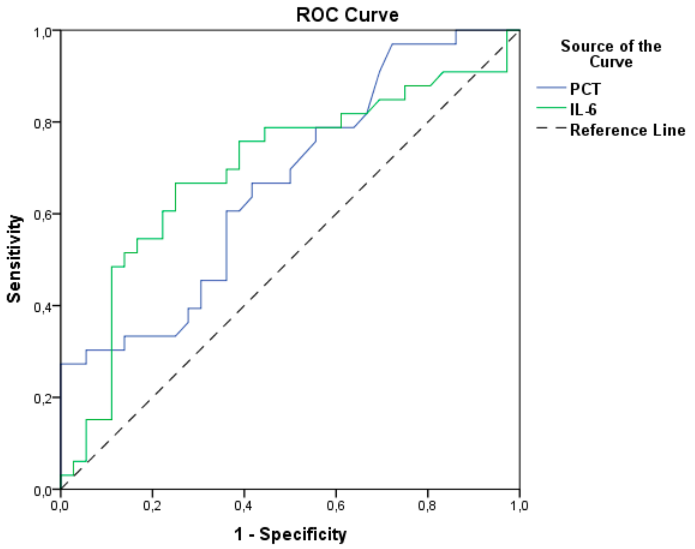

Area Under the Curve

Test Result Variable(s)	Area	p[b]	95% CI for AUC	
			Lower Bound	Upper Bound
**PCT**	**0.665**	0.019	0.538	0.792
**IL-6**	**0.696**	0.005	0.566	0.826

b. Null hypothesis: true area = 0.5

Supplement 3. Peak inflammatory markers in HAP-only and the No Bacterial Infection groups.

Variables peak values	HAP-only (n = 34)	NO BACTERIAL INFECTIONS (n = 36)	*p*-Value
CRP, mean (SD)	224.3 (±111.6)	154.7 (±85.8)	**0.007**
CRP change*, mean (SD)	83.8 (±103.9)	6.76 (±64.24)	**0.0004**
PCT, mean (SD)	8.6 (±21.3)	3.2 (±10.1)	**0.0005**
PCT change*, mean (SD)	7.6 (±21.41)	2.89 (±9.9)	**0.003**
IL-6, mean (SD)	1711.3 (±4089.1)	90.1 (±142.8)	**<0.0001**
IL-6 change*, mean (SD)	1473.87 (±4032.9)	29.9 (±75.1)	**0.001**
WBC $x10^9$, mean (SD)	20.1 (±9.2)	13.5 (±5.8)	**0.001**
WBC $x10^9$ change*, mean (SD)	7.46 (±7.55)	4.28 (±5.8)	**0.026**
temperature, mean (SD)	37.9 (±1.0)	37.4 (±1.0)	**0.022**
temp. change*, mean (SD)	0.7 (±1.4)	0.53 (±1.2)	0.503

* = peak value minus initial value

Supplement 4. The ability of peak values of PCT and IL-6 and their changes to predict HAP.

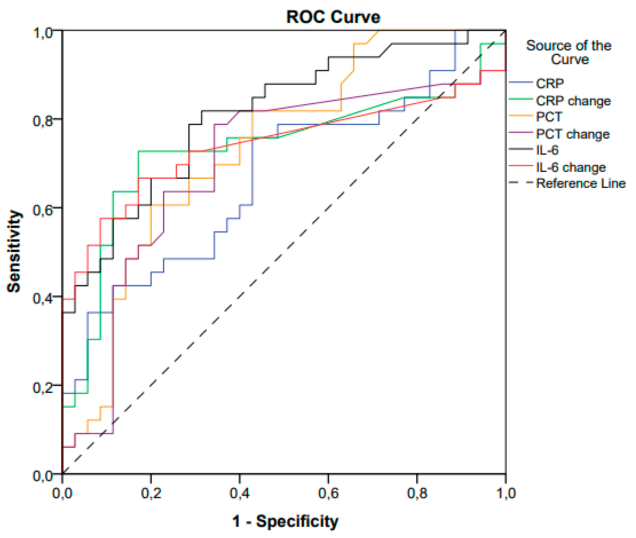

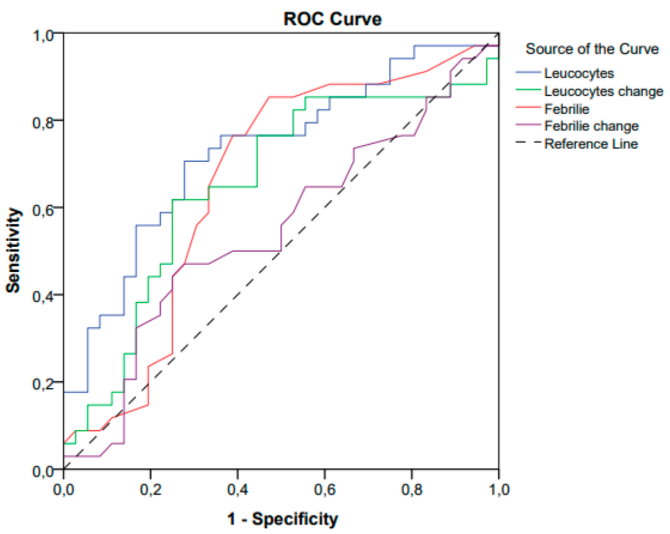

Area Under the Curve

Test Result Variable(s)	Area	p[b]	95% Confidence Interval Lower Bound	95% Confidence Interval Upper Bound
CRP (poor)	.671	.015	.541	.801
Change (fair)	.733	.001	.604	.863
PCT (fair)	.734	.001	.615	.854
Change (poor)	.697	.005	.564	.829
IL-6 (good)	.810	.000	.708	.912
Change (fair)	.741	.001	.613	.870

b. Null hypothesis: true area = 0.5

Area Under the Curve

Test Result Variable(s)	Area	p[b]	95% Confidence Interval Lower Bound	95% Confidence Interval Upper Bound
WBC (fair)	.730	.001	.610	.849
Change (poor)	.654	.026	.522	.787
Temperature (poor)	.659	.022	.526	.792

b. Null hypothesis: true area = 0.5
